# The mirror illusion: does proprioceptive drift go hand in hand with sense of agency?

**DOI:** 10.3389/fpsyg.2015.00200

**Published:** 2015-02-27

**Authors:** Daisuke Tajima, Tota Mizuno, Yuichiro Kume, Takako Yoshida

**Affiliations:** ^1^Applied Brain Science Laboratory, Department of Mechanical Sciences and Engineering, Tokyo Institute of TechnologyMeguro, Japan; ^2^Department of Informatics, Graduate School of Informatics and Engineering, The University of Electro-CommunicationsChofu, Japan; ^3^Department of Media and Image Technology, Faculty of Engineering, Tokyo Polytechnic UniversityAtsugi, Japan

**Keywords:** proprioceptive drift, sense of agency, sense of ownership, mirror illusion, vection

## Abstract

Vection can be regarded as the illusion of “whole-body” position perception. In contrast, the mirror illusion is that of “body-part” position perception. When participants viewed their left hands in a mirror positioned along the midsaggital axis while moving both hands synchronously, they hardly noticed the spatial offset between the hand in the mirror and the obscured real right hand. This illusion encompasses two phenomena: proprioceptive drift and sense of agency. Proprioceptive drift represented a perceptual change in the position of the obscured hand relative to that of the hand in the mirror. Sense of agency referred to the participants' subjective sense of controlling body image as they would their own bodies. We examined the spatial offset between these two phenomena. Participants responded to a two-alternative forced choice (2AFC) question regarding the subjective position of their right hands and questionnaires regarding sense of agency at various positions of the right hand. We analyzed the 2AFC data using a support vector machine and compared its classification result and the questionnaire results. Our data analysis suggested that the two phenomena were observed in concentric space, but the estimated range of the proprioceptive drift was slightly narrower than the range of agency. Although this outcome can be attributed to differences in measurement or analysis, to our knowledge, this is the first report to suggest that proprioceptive drift and sense of agency are concentric and almost overlap.

## Introduction

How does our brain know our body's position? In our daily lives, we rely on multiple cues from multisensory channels, such as vision, proprioception, and the vestibular system, to specify body position. However, in some situations, one channel can overwrite others in our perception: for instance, perception of the body's position can be altered easily by visual stimuli. When stationary participants view large patterns of optic flow simulating self-translation and self-rotation, they often experience illusions of self-motion known as vection (Fischer and Kornmuller, 1930; Seno et al., 2012). As visual stimuli simulating self-motion generate perceptual shifts in body position within the environment, vection can be regarded as the illusion of “whole-body” position perception induced by visual stimuli. In addition, visual stimuli, such as body parts, can modify the perception of body-part position. When participants viewed their left hands in a mirror positioned along the midsaggital axis while moving both hands, the mirror image strongly captured the left hand position and they hardly noticed the spatial offset between the hand in the mirror and the obscured real right hand (Holmes et al., 2004). This is termed the mirror illusion. This illusion encompasses a perceptual shift in body-part position from proprioceptive to visual feedback (that is, the image of the hand in the mirror) called proprioceptive drift.

Only a couple of previous papers have reported on the spatial properties that our brain uses to detect the discrepancy between visual and proprioceptive feedback of our body using proprioceptive drift by the mirror illusion. For example, Snijders et al. (2007) tested that discrepancy along with horizontal and depth lines on the table top during a reaching task and their results showed a maximal space of 5 cm for which participants did not notice the discrepancy between the real hand and the mirror image (namely, the visual feedback of their body part). However, there are still some problems with the estimation and visualization of this illusion. Since our hands can freely move in 2D and 3D space, analysis along only one dimension, such as the vertical, horizontal, and depth axes, is insufficient to examine the precise detail of the spatial properties of the illusion, especially regarding the relationship between visual and kinematic information of the arm and body. Another issue is how we can visualize this illusion in a 2D or 3D manner with a robust psychophysics method that is statistically satisfactory. The statistical method for psychophysics in past mirror illusion papers is optimized for experimentally controlled and limited conditions, not for natural conditions, where the participants can move their hand at their will.

Here, we propose a novel proprioceptive drift evaluation method to visualize the spatial discrepancy between visual and proprioceptive feedback to keep proprioceptive drift on a 2D plane by combining psychophysical procedures with machine learning using a support vector machine (SVM). To replicate previous findings regarding the mirror illusion, we investigated subjective body position using this method, both with and without visual feedback (i.e., mirror condition and blackboard condition), to clarify the effects of participant body movement on perception of an image of the body in a mirror (i.e., visual feedback).

In addition to proprioceptive drift, the mirror illusion is known to evoke two other types self-body sensations—ownership and agency (Gallagher, 2000; Holmes et al., 2004). Ownership is the sensation that a body part is one's own. Agency is the sensation that movement is self-caused. According to Gallagher's model, these two sensations are generated when actual sensory feedback from body movement matches predicted sensory feedback, according to the forward model of body movement. As feedback can be fundamentally multisensory, the model can include multisensory parts, such as visual, tactile, and proprioceptive feedback. However, Gallagher's model does not include this multimodal feature. As a result, it is unclear whether ownership and agency could be generated when different sources of multisensory feedback are inconsistent. With respect to the mirror illusion, proprioceptive feedback from the real hand would be spatially inconsistent with the visual feedback (i.e., the hand in the mirror). Even in this situation, the participant would perhaps feel clear ownership and agency as a result of the visual feedback in the mirror. In addition, some research has demonstrated generation of ownership and agency with temporal discrepancies between vision and other modalities (ownership: Shimada et al., 2009; agency: Farrer et al., 2008). This implies the potential for spatiotemporal discrepancies across multisensory feedbacks to maintain ownership and agency. This gives rise to the following questions: to what extent do multimodal discrepancies or proprioceptive drift and ownership and agency overlap? Do they go hand in hand? Are they the different aspects of the same process of self-body sensation or not? A principal component analysis result for multiple body-sensation test batteries shows that they can belong to the same cluster, suggesting that they can share the same information process to some extent (Jared Medina, personal communication, December 27, 2013). In contrast, a recent paper suggested that proprioceptive drift, ownership, and agency can be attributed to separable brain information processes (Rohde et al., 2011).

To answer these questions, we also investigated the spatial distribution of ownership and agency using questionnaires (common in the testing of these sensations), tailored for use with the mirror illusion on the midsaggital plane. Then, we investigated the overlap between the spatial distributions of proprioceptive drift and feelings of ownership and agency by comparing results. Through these experiments, we aim to clarify whether proprioceptive drift goes hand in hand with other body sensations, such as ownership and agency. In addition, we will discuss the potential application of the data for mechanical design of real-time control systems with self-body-like usability, and the objective, quantitative assessment of body sensations (especially assessing patients' self-body sensation changes during self-body control rehabilitation).

## Materials and method

### Participants

Ten graduate and undergraduate students from the Tokyo Institute of Technology were recruited as paid participants (all male, aged 19–28 years, Mean = 22.1, *SD* = 2.42). They all had normal or corrected-to-normal vision and were right-handed. All participants were naïve to the purposes of the study. The study was approved by the Ethical Committee of Tokyo Institute of Technology and all participants provided written informed consent prior to each experiment.

### Equipment

**Figure 1:**
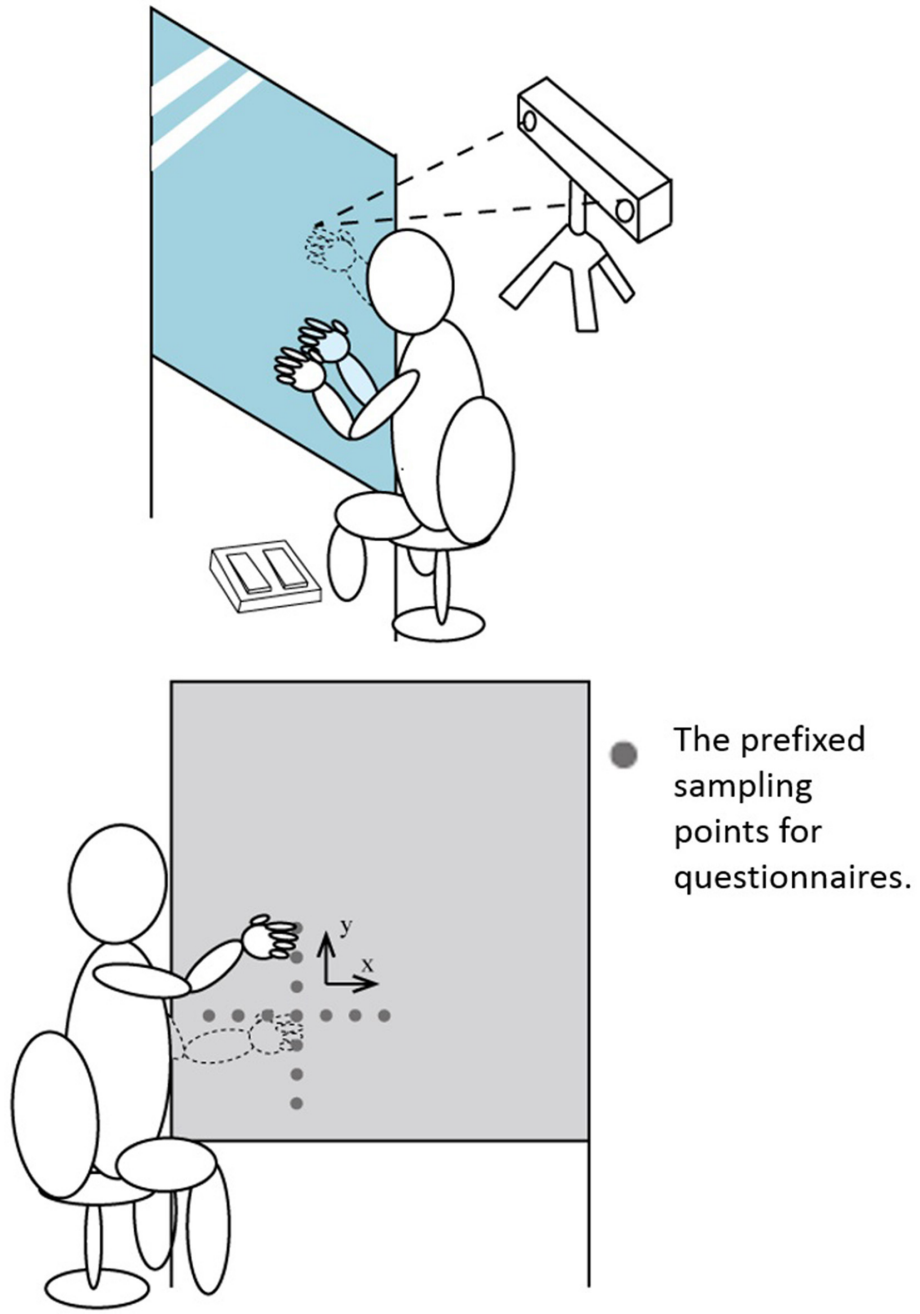
**The schematic representation of the experimental settings**.

A schematic representation of the setting is described in Figure 1. A 100 × 100 cm acrylic mirror was positioned vertically 60 cm above the floor, oriented parallel to the participants' sagittal axes with the reflective surface facing toward the participants' left. In the blackboard condition, a 100 × 90 cm matt acrylic blackboard was positioned in place of the mirror. In order to record the positions of participants' right hands, a position sensor (CyVerse, SLC-C02) was placed 1.0 m to the right of and facing the mirror. A custom-made retroreflective marker was attached to the participants' right index fingertips to relay their hand positions to the sensor. An infrared LED was attached at the top left of the reverse side of the mirror to record the relative positions between the fingertip marker and the edge of the mirror. A foot pedal (P.I. Engineering, Classic X-keys USB, and PS/2 Foot Pedals) was placed to the left of the mirror to collect participants' responses. Noise-canceling headphones (Bose, Quiet Comfort 3) were used to reduce the possibility that participants would hear the sound cue for the hand position.

### Experimental design and procedure

Two conditions were tested. In the mirror condition, participants saw their reflected left hand images in the mirror and could perceive the position of their right hands using at least two possible cues: (1) their proprioception and (2) the position of the right hand image in the mirror. The control condition included the blackboard, and participants perceived the position of their right hands using proprioception alone. The order of the conditions was counterbalanced across participants.

Prior to initiation of the experiment, participants were trained to tap both hands synchronously at roughly one cycle per sec (approximately 1 Hz) so that it is slow enough for the participants to observe the mirror image or their visual feedback, and to prevent them from executing this task only by their feed-forward system while ignoring the visual feedback. During the experiment, the experimenter monitored the timing of the participants' tapping and instructed them to reset their timing if it differed considerably from 1 Hz.

In the mirror condition, the participants were seated very near the mirror, so that their bodies almost touched it. They tilted their heads to the left slightly to look into the mirror. The participants' left hands were placed on the same side as the mirror. Their left index fingertips were approximately 30 cm vertically and 30 cm horizontally from the lower right corner of the mirror and 90 cm above the floor. Participants' left hands were fixed during the experiment. In contrast, their right hands were placed in a preferred position on the reverse side of the mirror. They could change the position of their right hands at will at the beginning of every trial. Participants were required to maintain the angles of both wrists. They were instructed to look at the reflection of the left hands in the mirror. After placing their right hands in new positions, the participants pressed the middle button of the foot pedal and started to tap the both hands synchronously at 1 Hz. They were required to tap more than six times. A previous paper reported that it requires at least 6 s visual stimulation to obtain sufficient amount of mirror illusion (Holmes et al., 2004) and six times tapping was almost equivalent to the 6 s stimulation since the tapping was 1 Hz. Therefore, we concluded that more than six times tapping was enough for our participants to observe sufficient amount of mirror illusion. After more than six taps, participants were required to answer two alternative forced choice questions (2AFC) by pressing the right or left button on the foot pedal. The question was, “Do you feel that both hands are in the same position?” When the foot pedal was pressed, participants' responses and the positions of their right hands were recorded, and a beep sound served as a sign to change the position of their right hands for the next trial. This process was repeated for a maximum of 200 trials per condition.

In the blackboard condition, the procedure was almost the same as that of the mirror condition, except that participants were instructed to look at the position of the left hand's reflection when the mirror was used in place of the blackboard.

In a separate experimental session, we also measured sense of ownership and agency with respect to the hand in the mirror, using a common 16-statement questionnaire in the mirror condition to replicate previous findings and compare results to proprioceptive drift data. Table 1 shows the questionnaires used in Japanese and their English translations. From top to bottom, four statements referred to ownership (e.g., “I felt as if I was looking at my own right hand.”), and four described the sensation related to agency (e.g., “I felt as if I was causing the movement I saw as my right hand”). The remaining eight statements were control statements, with four for ownership and four for agency (e.g., “It seemed as if I had more than one right hand” and “I felt as if the hand in the mirror was controlling my will”). These questionnaires were adapted and translated into Japanese from questionnaires used in a rubber-hand illusion experiment (Botvinick and Cohen, 1998; Kalckert and Ehrsson, 2012).

**Table 1:**
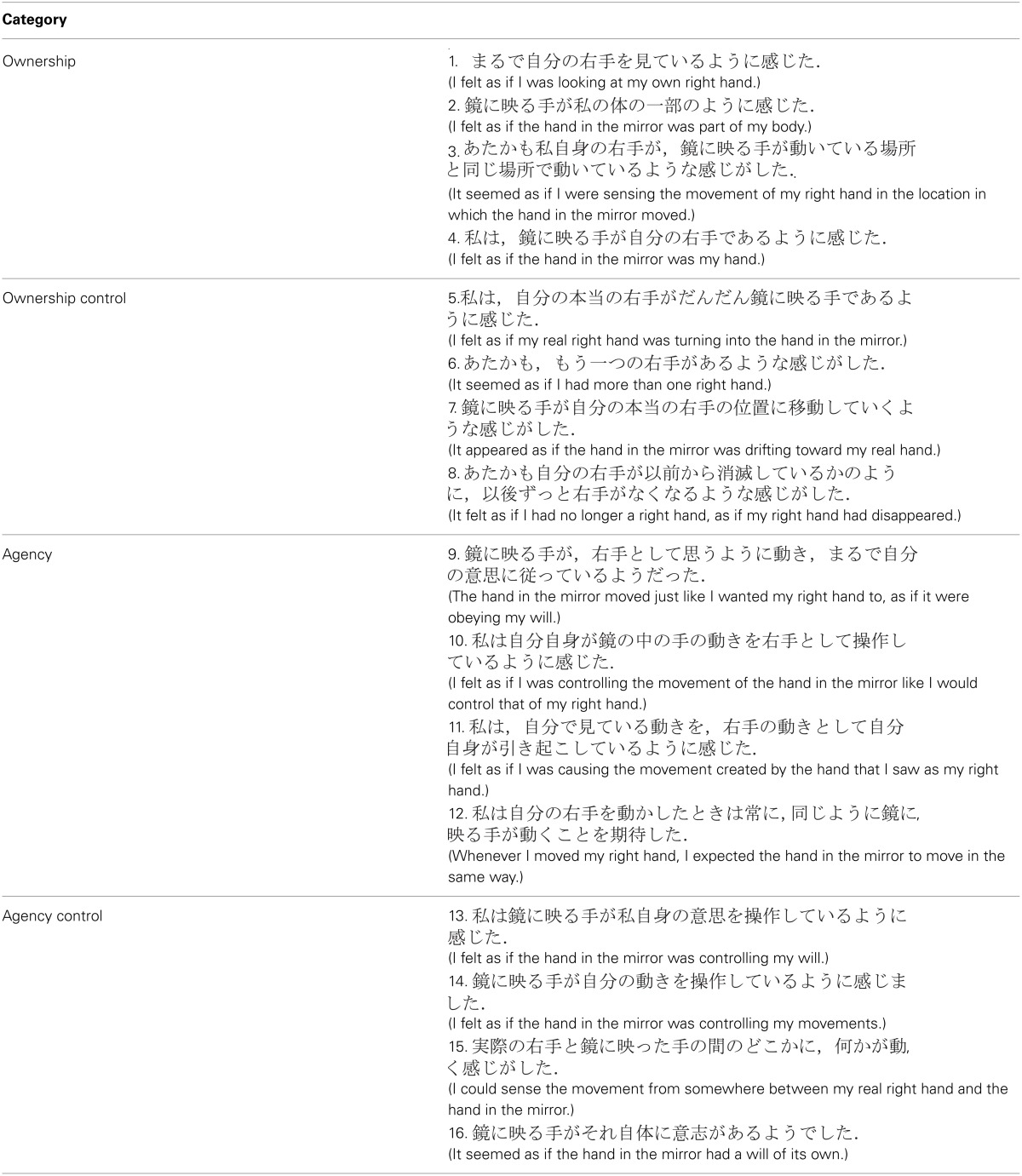
**The questionnaires consisted of 16 statements classified into four categories in Japanese and English**.

In the experiment measuring proprioceptive drift, the participants could choose the position of their right hands freely. In contrast, to measure feeling of ownership and agency, 13 prefixed right hand positions were examined (Figure 1). These points were arranged every 7 cm up to ±21 cm from the original position, both vertically and horizontally. The participants were required to respond to the questionnaires after tapping both hands synchronously six times at pre-fixed positions. They were not required to press the foot pedal in this session. Participants responded to questionnaire items using a 7-point Likert scale with ratings ranging from “−3” (totally disagree) to “+3” (totally agree) and “0” indicating neither agreement nor disagreement (“uncertain”).

### Data analysis

**Figure 2:**
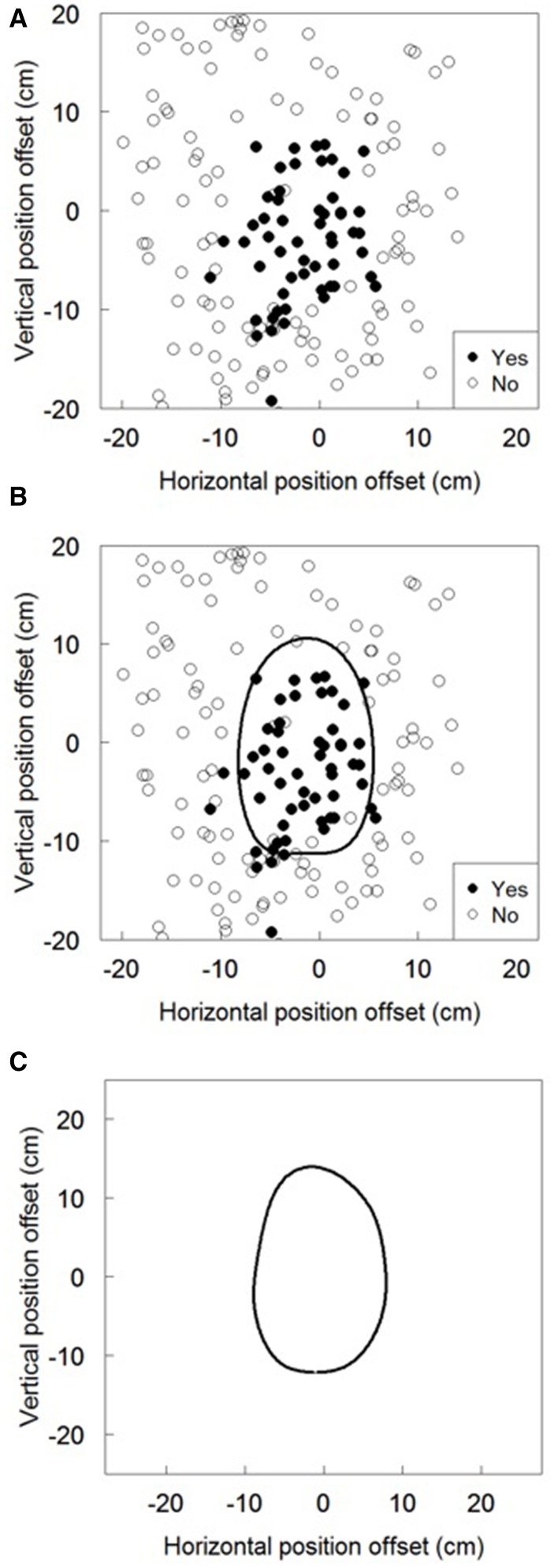
**The data analysis flow**. **(A)** is the raw plot of one participant's responses and right hand positions. **(B)** is the result of the border analysis using support vector machine. **(C)** is the average of the border across the participants.

Figure 2 shows the flow of the data analysis for proprioceptive drift. Figure 2A is the raw plot of one participant's data, Figure 2B is the result of the border analysis using support vector machine (SVM), and Figure 2C is the average across participants. Analysis was two staged; the first was within and the second was between participants. The first stage involved subjective position sensation for the right hand, with within-participant analysis conducted to determine the area in which participants did not notice the spatial gap between their visual and physical bodies. Between-participant analysis was conducted to obtain an average of the spatial area provided by participants' “Yes” responses.

In the within-participant analysis, an SVM was used as the classifier (Karatzoglou et al., 2004) to extract the borders of participants' responses. The SVM provided a probabilistic model of each participant's response in 2D space, trained using the data of their responses (Yes/No) and the position of the right hand at the time of response. Bishop (2006) provided an explanation for the algorithms of the classifier (See Chapter 7 in Bishop, 2006). The area in which the *p*-value of the participant's “Yes” response was estimated to be over 0.5 was defined as the area in which the participant did not notice the spatial gap between body image in the mirror and proprioception of the right hand. The kernel used for the SVM was the commonly used radial basis function kernel. In order to build the static model, the virtual participant's responses were added. The virtual responses were all “No” responses, and its positions are described in Table 2, showing that participants could not reach their right hands.

**Table 2:** **The positions of the virtual response in the SVM analysis**.

**No**.	**Position**	
	**X (cm)**	**Y(cm)**
1	30	30
2	15	30
3	0	30
4	−15	30
5	−30	30
6	30	15
7	−30	15
8	30	0
9	−30	0
10	30	−15
11	−30	−15
12	30	−30
13	15	−30
14	0	−30
15	−15	−30
16	−30	−30

After running the above analysis for each participant's data, we averaged the result across participants. As it was difficult to average the border of the “Yes” and “No” response area estimated by *p*-values for the participants' responses in 2D space, we attempted two averaging methods. One method was to average the *p*-values for the participants' responses in 2D space, which was the method used prior to estimating the border. The other method was to average the area size, which was used after estimating the border.

All data were assessed for a normal distribution using the Shapiro-Wilk test (*p* > 0.05), and the appropriate non-parametric tests were applied when one or more of the corresponding data sets failed to meet the criteria for normal distribution. Area size data were not normally distributed due to participant variance; therefore, a Wilcoxon signed-rank test was used for pairwise comparison. The questionnaire data were not normally distributed; however, we performed a Two-Way repeated-measures ANOVA to analyse the questionnaire data, as there was no non-parametric substitute for this analysis. The results of the Mauchly's sphericity test were not significant (*p* > 0.05) for the questionnaire data. Therefore, we did not use the Greenhouse-Geisser corrections.

The analysis comparing area size and questionnaire data was conducted using SPSS (version 21, IBM Corp., Armonk, NY, USA). Asterisks in plots indicate significance levels: ^*^p < 0.05.

## Results and discussion

**Figure 3:**
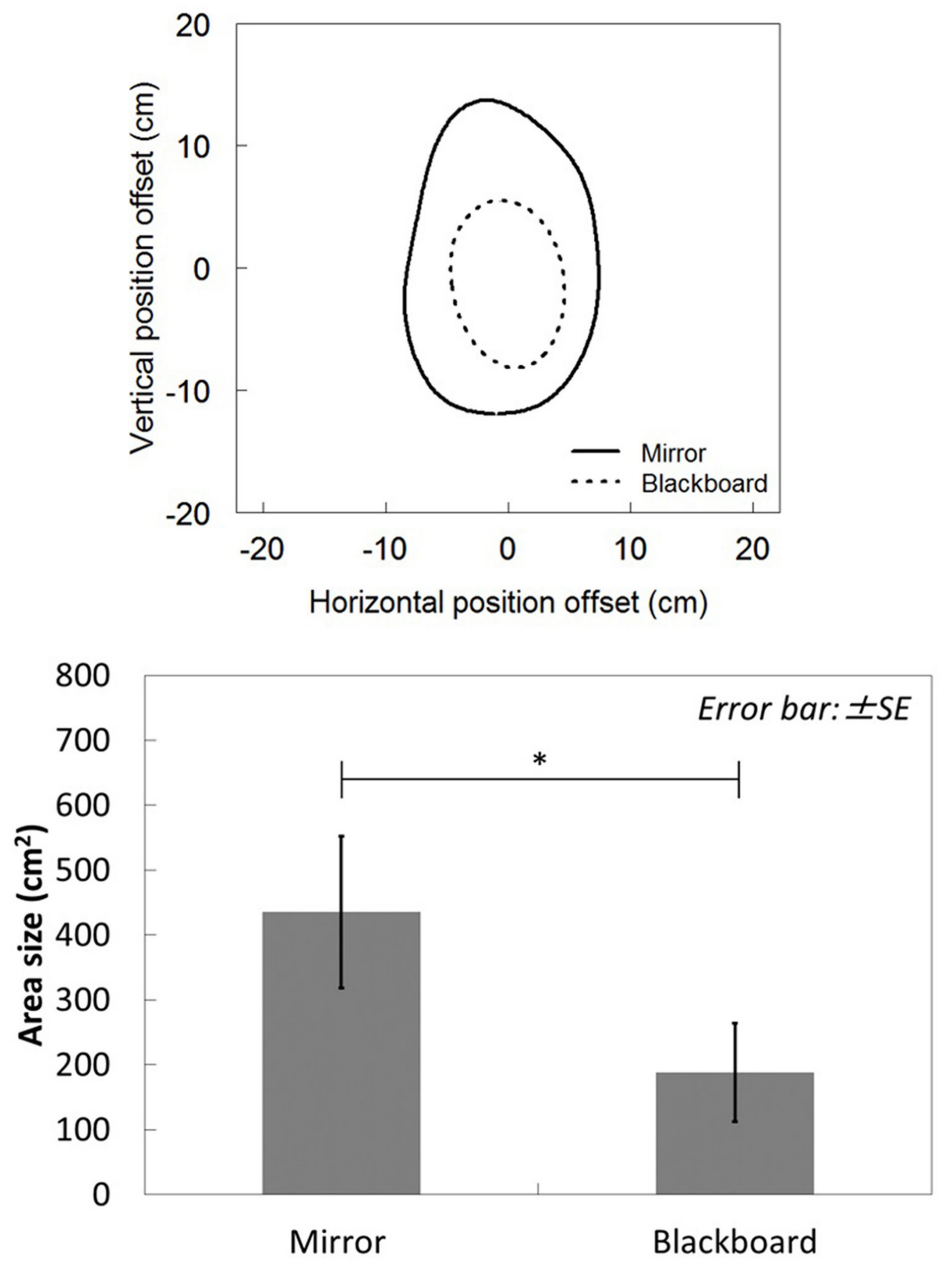
**The comparison of area shape and size between the mirror and blackboard conditions**. ^*^p < 0.05.

Figure 3 shows the comparison of area shape between the mirror and blackboard conditions. The origin represented the position of the left index fingertip, or the position of the visual feedback in the mirror condition. The vertical and horizontal spatial offset between the left and right hands formed the axes. In both conditions, as their right hands approached the origin, the participants tended not to notice the spatial gap between their left and right hands. However, in the mirror condition, the shape and size of the area clearly differed from those in the blackboard condition (Wilcoxon signed-rank test: *Z* = −2.803, *p* = 0.005). This result was consistent with previous findings regarding the mirror illusion (Holmes et al., 2004), suggesting that our method showed the visual capture effect successfully. Based on our results, the offset between visual and proprioceptive feedback afforded up to approximately 10 cm to maintain proprioceptive drift.

Our visualization method revealed that the required offset to maintain proprioceptive drift was approximately 10 cm. This distance was larger than the value measured in previous research using the rubber-hand illusion paradigm (Tsakiris et al., 2010; Rohde et al., 2011; Kalckert and Ehrsson, 2012). In this regard, two factors may have accounted for this difference. The first was the reality of the hand's visual feedback. According to the study conducted by Tsakiris et al. (2010), the appearance of a hand image would play an important role in evoking feeling of ownership and proprioceptive drift. Compared to the rubber hand, the hand image in the mirror matched the real appearance of a hand almost perfectly. This may have emphasized the effect of visual capture, increasing the offset value.

The second factor was the movement of the hand. In the rubber-hand illusion paradigm, the participant's hand and the rubber hand received synchronous strokes of a paintbrush. These stimuli provided visual and tactile feedback from the hand to evoke ownership of the rubber hand. In contrast, under the paradigm used in this research, the participant moved both hands synchronously while viewing the hand in the mirror, positioned along the midsaggital plane. The main difference between the rubber hand illusion and the mirror illusion was whether the participant received feedback from their voluntary body movements or not. We speculate the difference between the voluntary and involuntary body movement can be attributed to the difference of the involvement of “attention” to enhance the saliency of controlled objects in the visual field. Kobayashi and Yoshida (2014) found that moving objects or visual feedback that matched ongoing action or hand movement can be found faster, as in the pop-out phenomenon in visual search by higher-order visual features, suggesting the involvement of attentive processes in the perception of self-controlled objects such that the saliency of the object is enhanced quickly with minimum attentive demands. If the same finding can be applied to moving hand images in the visual field, then the hand image in the mirror would attract the participant's attention relatively quickly with minimum effort. In contrast, in the rubber hand illusion paradigm, the rubber hand does not attract participants' attention—rather, the participant must voluntarily pay attention to the rubber hand. Such differences in participants' attentive states may contribute to the time course and enhancement of visual capture between voluntary and involuntary object or image control. The relationship between the attention and detectability of multimodal discrepancies would be the next matter to discuss.

In the mirror condition, vertical (altitude) offset between visual and proprioceptive feedback appeared larger than horizontal (radial) offset. This result supported direction-dependent differences in visual-proprioceptive integration (van Beers et al., 1999, 2002). Snijders et al. (2007) discussed similar differences. They investigated the effect of visual capture by measuring reaching error when participants viewed the hand in the mirror as a substitute for a real hand. Their results showed that azimuthal (directional) errors were significantly larger than radial (distance) errors. This result also supports direction-dependent differences. Our findings imply that the radial information of the hand position was handled more precisely relative to the azimuthal and altitude information. One possible reason for this eclipse-shaped proprioceptive drift is differences in the proprioceptive sensibilities of the elbow and shoulder joints. When moving one's hand in a radial direction, the elbow joint angle increases relative to that of the shoulder joint. In contrast, when moving the hand in an altitudinal direction, the shoulder joint angle increases relative to that of the elbow joint. This difference may generate direction-dependent differences in visual proprioception. In this study, we did not measure joint angles and potential correlating factors such as power of muscles connected to the joint or sensations from the joint like painfulness. Further research is required to explore these factors.

**Figure 4:**
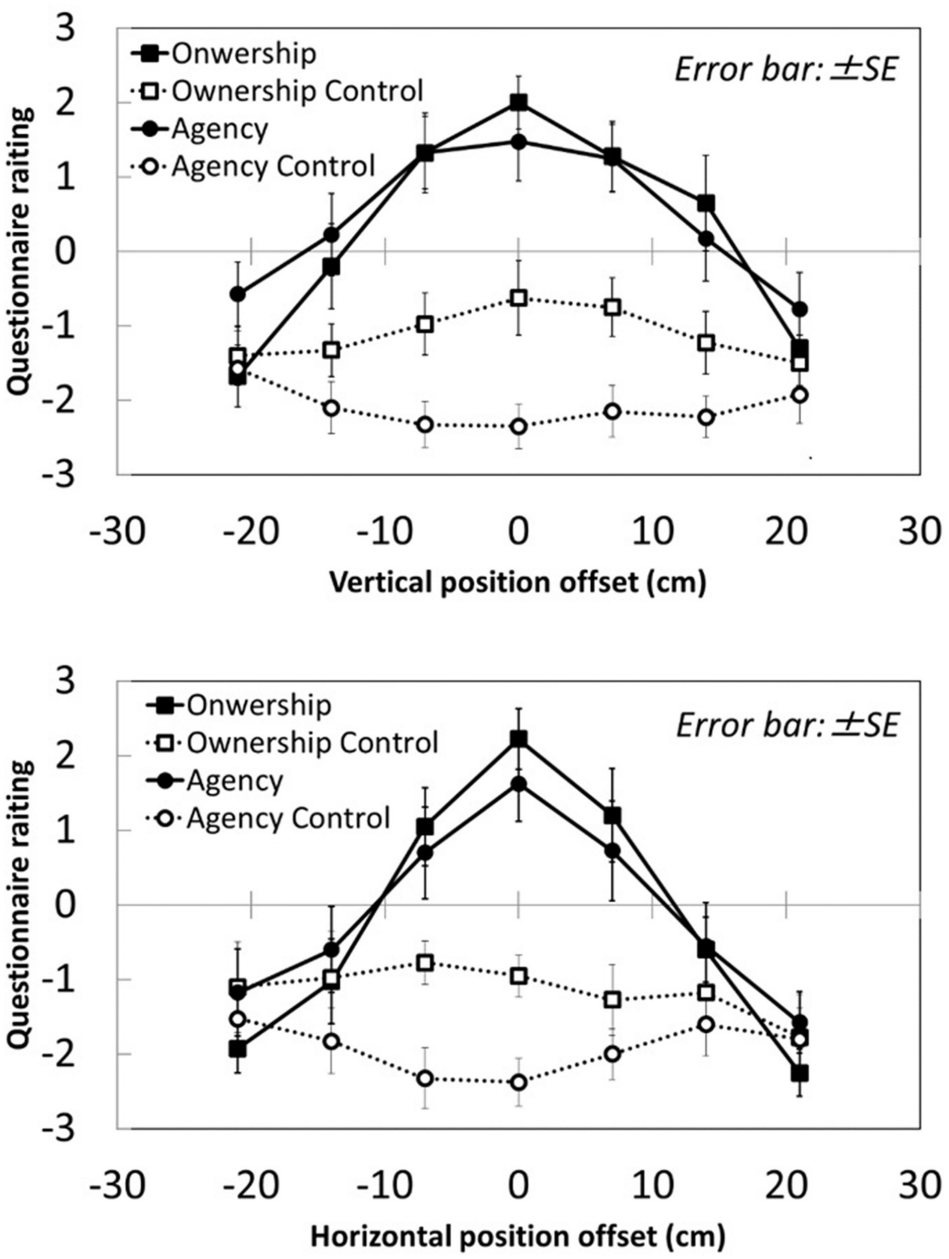
**Questionnaire results**.

Figure 4 shows questionnaire results about ownership and agency at each sampling point. The axes show vertical and horizontal spatial offset between the hand in the mirror and the obscured real hand. The plots and lines represent differences between questionnaire categories; the black square, white square, black circle, and white circle represent ownership, ownership control, agency, and agency control, respectively. Ownership and agency scores were highest at the origin relative to the positions in vertical and horizontal directions. The scores for control statements showed little difference between positions. A Two-Way repeated-measures ANOVA comparing questionnaire categories and positions revealed that the main effects of category and position were significant [Horizontal: Category: *F*_(3, 27)_ = 11.12, *p* < 0.001; Position: *F*_(6, 54)_ = 10.27, *p* < 0.001; Vertical: Category: *F*_(3, 27)_ = 24.21, *p* < 0.001, Position: *F*_(6, 54)_ = 7.298, *p* < 0.001]. The interactions between category and position were also significant [Horizontal: *F*_(18, 162)_ = 9.42, *p* < 0.001; Vertical: *F*_(18, 162)_ = 8.00, *p* < 0.001]. These results indicated that less distance between the hand in the mirror and the real obscured right hand was associated with greater feeling of ownership and agency with respect to the hand in the mirror. Our data show that ownership and agency were evoked at up to ±10 cm vertically and ±15 cm horizontally from the origin in the mirror illusion.

**Figure 5:**
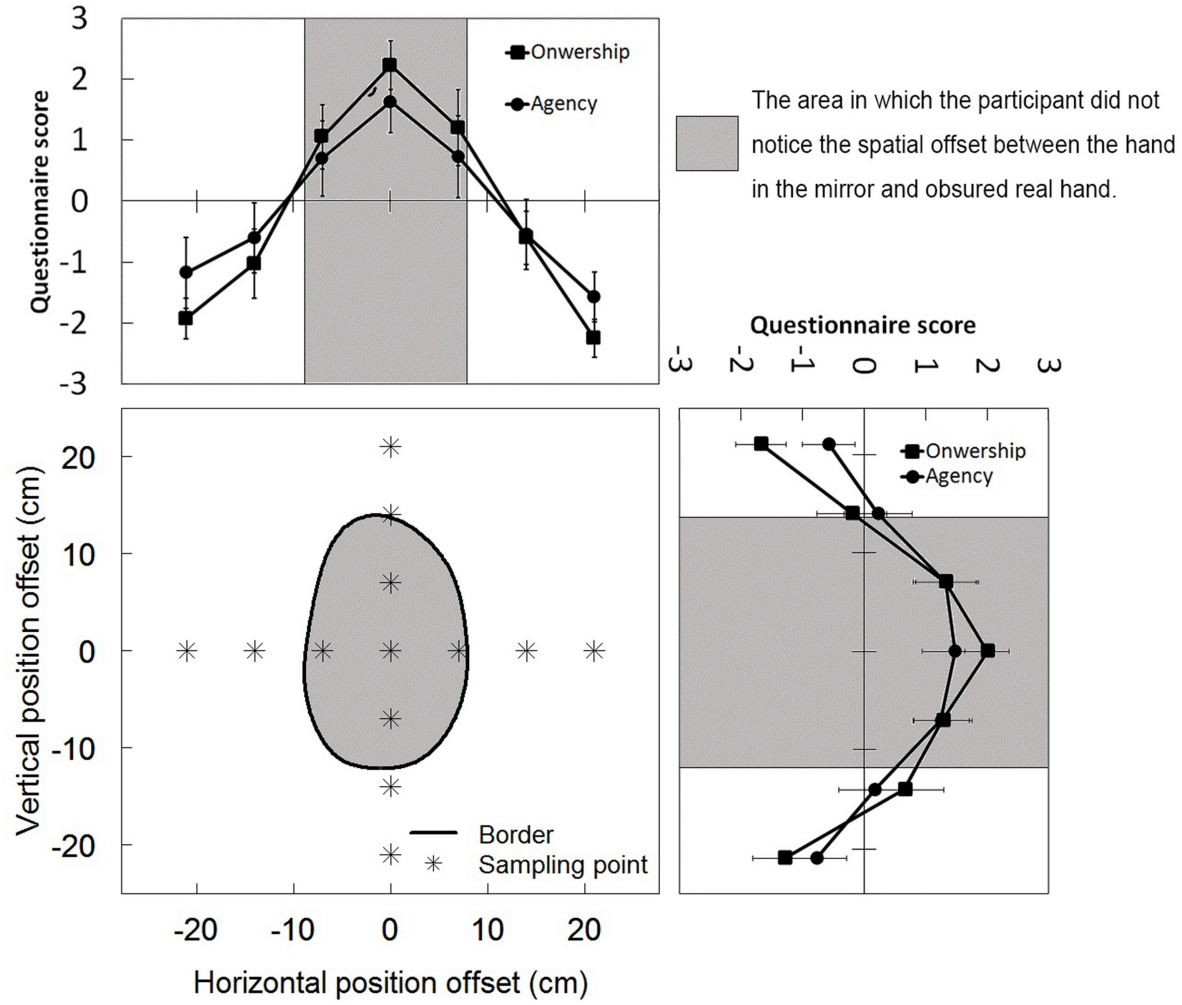
**The comparison between the classification and questionnaire**.

Figure 5 shows the comparison between the classification of 2AFC data and the questionnaire results. The highest questionnaire scores for sense of agency and ownership were in almost the same position in the center of the area in which the participant did not notice the spatial gap between the hand in the mirror and the obscured real hand. The zero crossing point of the scores for agency and ownership was in almost the same position relative to the border of the area in a horizontal direction; however, in a vertical direction, it differed slightly relative to the position of the border. These results suggest that the offset between visual and proprioceptive feedback maintaining proprioceptive drift overlapped almost perfectly with the offset, evoking feelings of ownership and agency as a result of visual feedback from the body.

Our findings showed that the spatial offset between visual and proprioceptive feedback evoking proprioceptive drift was similar to the offset required to maintain ownership and agency. This result indicated that the same integration or matching processes between visual and proprioceptive feedback could be used to evoke proprioceptive drift, feeling of ownership, and agency. However, the overlap of underlying mechanisms between proprioceptive drift and ownership and agency remains unknown. In this regard, Rohde et al. (2011) provided evidence of dissociation between feeling of ownership and proprioceptive drift in the rubber-hand illusion paradigm. They found that proprioceptive drift was observed between the asynchronous rubber hand and the real hand, which was used in the condition that did not generate ownership. The differences between the brain mechanisms underlying proprioceptive drift, ownership, and agency are an important issue in neuroscience research. In contrast, the aim of the current study was to describe the spatial overlap area between these phenomena in the strategic design of controlling sensations. On this point, it may be said that this purpose was successfully accomplished.

In this study, we investigated the spatial discrepancy between visual and proprioceptive feedback that maintains the sensations about self, ownership, and agency. In the vision experiment, we investigated whether the visually observed body is attributed to the “self” or not. This distinction between “self,” “non-self,” or “others” has been the subject of recent extensive discussion on brain mechanisms, and is called the “social brain.” However, the distinction between the self and non-self attributions based on some perception signal or event has been repeatedly discussed in vision science research (especially in the research area of motion perception), specifically relating to how the brain discriminates between retinal motion caused by the observer's body movement (including eye movement) and motion caused by other agents, such as objects or environment. Vection, the illusion that results from the participant misjudging whether the body or the environment is moving, is a typical attribution problem tested in perception research. Precise comparison between the perception-driven self and the social brain-driven self (including self-body studies) will provide insight into the relationship between our conscious visual experience (or awareness) and motor actions. In classical vection studies, whole-body position movement can be observed in the opposite direction of the visual stimulation. However, we can find a few reports on vection in the same direction as the visual stimulation, called inverted vection (Nakamura and Shimojo, 2000; Saito and Sakurai, 2014). The precise underlying mechanisms of this type of vection are not currently clear. However, it is likely that inverted vection can be attributed to some brain process involving the grouping of surrounding space and one's body, which then move together in the same direction in relation to another space—for example, a driver's space in the car, the driver's body, and the space or environment outside the car when driving forwards (Tajima et al., personal communication, 2013). In this case, vection or whole body position detection relies on a process utilizing the space occupied by the body (e.g., the space in the car) and how it is moving together with “us,” rather than the surrounding environment. In this case, the vection direction can be the same as the visual stimulation directions when it appropriately meets some requirements. Thus, we postulate that the self-body part image is integrated with outer visual space information as if the latter belongs to our “self.” With this process, we can postulate a variety of subprocesses involving only the self-body image or inner model during movement, although we did not examine this idea in the present report, and the speculation and expectation for the incoming visual feedback based on it. One future direction for inversed vection research could be to examine this action-feedback information-grouping process in relation to self-body sensation factors, such as sense of agency and ownership. This would be a relatively new research topic wherein we could test integration of intentional body movement and incoming visual information or action-feedback causality perception, thus confirming the results of some recent studies (Farrer et al., 2013; Kawabe et al., 2013).

Our initial motivation to run this type of research is to apply it for the mechanical design. In our daily lives, we scarcely pay attention to the spatial discrepancy between modalities. In contrast, this spatial discrepancy can be found when we design and manipulate real-time control systems, such as remote control systems, robotic surgical systems, or virtual reality systems. Robotic surgical systems, for example, receive the user's hand movements as the input for operating the robotic arm. Then, the system provides the user with the consequence of robotic arm movement on a monitor. The visual feedback of the user's body is substituted with the robotic arm, that is, the visual object in the monitor. In the case of virtual reality systems, visual feedback is substituted using the virtual body depicted on a monitor. In these systems, the distance between the user's body and the visual feedback or object that can be substituted to the body is often unavoidable due to physical constraints of the mechanical design. For instance, the position of the virtual hand on the monitor does not share the same space as the user's real hand or the input device (such as a joystick). Therefore, without some appropriate calibration between the user's hand and the monitor, the locations of the visual and proprioceptive feedbacks are physically distinct. Despite this situation, if the user is unable to detect the spatial discrepancy between visual and proprioceptive feedback and they feel that their real hand position is at the visual feedback position, their usability and experience during operation of the system would not be so different from that for our own natural arm and hand. The precise detail of this type of real time controlling system design and this type of human sensations derived from several phenomenon including mirror illusion is still under debate along with “embodiment” issues on the possibility to replace our real body feedback to the virtual or mechanical one while keeping natural, intuitive sensations and usability (e.g., Rosén et al., 2009). In addition, VR researchers do not always carry out this type of calibration between vision and haptic information since they know that users do not notice the small spatial offset between modalities. Not all the visuo-haptic virtual reality systems and real-time controlling systems are intended for the self-body sensations tested here. However, our result and the assessment method can be the first step to show this type of room to allow us to ignore the visual and haptic perfect overlap while keeping their natural sensations.

According to research on self-body sensations, if the relationship between action and feedback meets certain criteria, self-body sensations can be felt despite the absence of a physical link between brain and body. Some clinical phenomena and psychological findings support this proposal. In clinical cases, phantom limb sensation and alien hand syndrome are powerful evidence indicating that the physical linkage between brain and body is not a matter of self-body sensation alone. The former phenomenon is where a patient without a limb still feels the missing limb (Ramachandran et al., 1995). The latter is a phenomenon where patients feel as if their limb is that of another person (Banks et al., 1989). In psychological cases, the mirror and rubber hand illusions (in which the participant can feel self-body sensations for a rubber hand by synchronous paintbrush strokes to their obscured hand and the rubber hand) support the relationship between action and feedback, and allude to the matter of different modalities in feedback. According to Gallagher's model and other research, the generation of self-body sensation seems to depend on whether or not sensory feedback occurs with action or other modalities in space and time. Based on this idea, it is possible to create self-body sensation for an object other than one's body by optimizing the action–feedback relationship in space and time in some multimodal manner. The possibility of eliciting self-body sensations with manipulation of a real-time control system may lead to important developments. In other words, it can inform techniques for projecting the user's “self” into a static or moving visual object, such as the virtual avatar and remote control robot. In addition, if users could project their self-body sensation onto the visual feedback or object on the monitor (as in the avatar body and robotic arm), it becomes possible to obtain an intuitive sense of control during operation—namely, it would be as if they were controlling their own body, or “diving into” the mechanical or virtual body. This can lead to the establishment of design rules for the self-body (such as intuitive usability), simply by optimizing human body movement and its feedback of human body processes. If we apply our current findings to such design rules, we can conclude the following. If the position of visual feedback is within the estimated area (see Figure 3) when the actual hand position is at its origin, the operator could cannot notice the spatial gap across the modalities and can maintain self-body sensation. However, our finding is limited to when the visual feedback is highly similar to that of a human body, with almost zero temporal delay and an exceedingly high-resolution image. Compared to the human body, the visual feedback in artificial systems (such as real-time control systems) has various physical transformations, such as shape, color, position, and time lag. Therefore, whether our findings can generalize to these cases may be an interesting topic for future research.

Our finding may provide an important contribution to the field of rehabilitation. In self-body rehabilitation, treatments to improve motor function and pain are often carried out for patients with missing or paralyzed limbs. Although these treatments can help patients recover functioning in order to perform their daily life, the treatments do not guarantee full to recover the feelings of ownership and agency for those limbs. This type of treatment has just begun in recent years and the most efficient method of treatment is still under discussion. Unlike physical treatment with surgery, the treatment of ownership and agency is based upon the treatment of the patient's subjective experience. In general, subjective experience differs across patients. Moreover, since the estimation of subjective experience relies almost solely on the qualitative method (such as the questionnaires) it is difficult to determine the reason why patients are missing ownership and agency for their limbs after treatment. Therefore, in order to test why this is happening, an objective and quantitative method is needed. Our finding could lead to the provision of psychophysics data upon further investigation.

In conclusion, there were two main findings in this study. First, we successfully visualized the precise spatial offset between visual and proprioceptive feedback by maintaining proprioceptive drift on the midsaggital plane. This offset afforded up to approximately 10 cm between the proprioceptive and visual feedback. Second, despite the difference in measurement, we showed partial spatial overlap between proprioceptive drift and feelings of ownership and agency. This is the first report suggesting that spatial overlap between these two phenomena were almost perfect. We are interested in whether this finding can be replicated with real-time control systems, where the offset between visual and proprioceptive feedback is within the range that maintains proprioceptive drift and self-body-like usability. These types of study would help to understand our brain mechanism that produce an experience of “self” and allow us to discuss the action-feedback relationship to position or maintain our “self” in a manner that it's general from the artificial body to the natural body.

### Conflict of interest statement

The authors declare that the research was conducted in the absence of any commercial or financial relationships that could be construed as a potential conflict of interest.
